# Investigating the Usage Patterns of Park Visitors and Their Driving Factors to Improve Urban Community Parks in China: Taking Jinan City as an Example

**DOI:** 10.3390/ijerph192315504

**Published:** 2022-11-23

**Authors:** Deyi Kong, Zujian Chen, Cheng Li, Xinhui Fei

**Affiliations:** 1 College of Landscape Architecture and Art, Fujian Agriculture and Forestry University, Cangshan District, Fuzhou 350002, China; 2 College of Arts, Shandong Jianzhu University, Jinan 250101, China

**Keywords:** community parks, urban green space, mixed methods, park usage, driving factors

## Abstract

Urban community parks have significant benefits for city residents, both physical and spiritual. This is especially true in developing countries, such as China. The purpose of our study is to describe the current situation of the community parks in five main districts of Jinan City while recognizing features of the community parks that influence usage patterns. Our study also means to determine the desired improvements of visitors that promote access to and use of community parks on the basis of the Chinese context. We conducted a survey among 542 community park visitors and obtained valid responses. The findings of respondents show that community parks are mostly used by people over 55 years (34.7%) and children under 10 years (23.6%). The main motives for using community parks are for exercise (24.2%) and to socialize with others (21.6%). The majority of respondents (65.7%) rated the community park as satisfactory and considered only a few improvements needed. Regarding the desired improvements, numerous respondents mentioned adding more physical training facilities (13.3%) and activity areas (7.6%), as well as emergency call buttons in areas frequented by children and older people (7.6%). Furthermore, most of the respondents (79.9%) indicated that they would like to use the community parks more frequently if there is additional progress to make the parks more attractive, cleaner, and friendlier. These results can help park designers, government agencies, and community groups to provide the planning and design strategies for community parks to promote their upgrading in China.

## 1. Introduction

Urbanization is advancing worldwide, and by 2050, 68% of the world’s population is projected to live in urban areas [1]. This rapid urbanization has led to an accelerating decline in urban green space [2,3]. Therefore, the planning and design of urban green infrastructure (GI) is attracting increasing global attention, which is a network in the city of interconnected green spaces consisting of patches, corridors, and matrixes to protect the diverse interests of human beings, biodiversity, and habitats of various species [4,5]. In the current situation of accelerated urbanization and surging urban population, plenty of researchers emphasize the introduction of community parks or numerous minor urban green spaces as a critical “patch” of the urban green infrastructure, which can serve the interests of numerous communities [6,7,8].

On 1 June 2018, the Chinese Ministry of Housing and Urban-Rural Development issued a standard for urban green space classification [9], defining “community park” as an independent area with basic recreation and service facilities and its urban green spaces developed mainly for residents of a particular community to conduct daily recreational activities on 1–10 hectares of land. Community parks have myriad benefits for residents and the city’s environment. In terms of residents, they provide more opportunities for people to connect with nature, provide a space for social engagement and exercise, reduce obesity, depression, and anxiety, and positively impact people’s bodies and minds [10,11,12]. In terms of the environment, they have been called the “lungs of the city” because they help provide fresh air, increase biodiversity in the area, improve the microclimate, and mitigate the heat island effect [13,14,15].

Over the past decade, urban green infrastructure (GI), such as rainwater gardens, linear green spaces, and wetlands, have attracted the attention of numerous researchers, with large public parks being the most studied [16,17,18]. However, studies on community parks remain limited [19]. Most of them have targeted specific people and content, such as older adults and children [20,21], and several studies have provided valuable insights into residents’ preferences for community parks in different thematic styles and how landscape features influence people’s access to, interaction with, and satisfaction with the parks [22,23,24]. Dunton et al. [25] concluded that although a community park area is small, a large number of them are in a city, and they are the most underrated but potentially valuable ecological resources of urban GI. Furthermore, in recent years, urban densification, particularly in developed countries (such as Germany and The United States) and in developing countries in transition (such as China and Malaysia), has strained limited land resources and reduced the opportunity to create larger green spaces in cities [26]. The loss of green spaces has reduced residents’ access to nature. This affects the frequency of use, where longer distances reduce the frequency of use, harming public health [27,28,29]. As a result, numerous cities need to pay more attention and establish more small urban green spaces, such as community parks located around living and working environments, to improve urban ecology, enhance urban quality and improve the quality of life for residents [30,31]. This is vital as community parks are the most exposed green infrastructures in urban areas, with a well-designed and managed community park increasing access to nature and opportunities for health and fitness for diverse communities [32,33].

Even though community parks have been created to benefit urban populations, especially within developing countries such as China, research on their usage and perception is lacking. Currently, in urban construction in China, community park construction is mainly contracted to the design company, which simply passes the designer’s landscape awareness to the visitors, and the visitors can only be given local or forced changes after obtaining the right to use the park [16,18,34,35,36,37]. Shinew et al. [38] concluded in his study of American urban parks that the subjective initiative of the visitors serves as the constructing and operating logic of community parks. However, the current Chinese community parks violate this logic. Research on visitor usage patterns of and desired improvements to urban community parks is vital, as those small urban green spaces such as community parks that do not meet most visitors’ expectations could be easily replaced with other land uses [39,40]. More research on community parks is needed to determine their importance to city residents and to encourage their construction. China is in the midst of an urban renewal movement. The urgency of community park research stems from the fact that it can effectively help residents meet their demands for a better life. It is necessary to study the usage patterns of park visitors and their driving factors to improve urban community parks to guide the revitalization and construction of community parks [41,42].

In addition, research on community parks is mainly focused on North American or European contexts currently [43,44,45]. Sparse research has been performed on the usage patterns of community parks, especially in the Chinese context. It is essential to diversify this research because people’s outdoor activities are often determined by their cultural backgrounds, and there are wide differences among countries in beliefs, politics, and lifestyles [46,47,48]. The findings and conclusions of studies conducted in North American or European contexts do not apply to China, which is relatively integrated in terms of ethnicity, culture, and religion. Therefore, an understanding of the usage and perception of community parks in China context is necessary [49,50]. In this study, we focus on community parks following the definition given by the Chinese Ministry of Housing and Urban-Rural Development [9]. To the best of the author’s knowledge, there is no study of visitor usage patterns and desired improvements in community parks in Jinan City or in any other region of China. Our study is formulated to fill the gap of knowledge in this area through the following questions: (a) Who uses community parks? (b) How do park visitors use the community parks? (c) What are the visitors’ desired improvements to the community parks in Jinan City?

## 2. Methodology

### 2.1. Study Area

**Figure 1:**
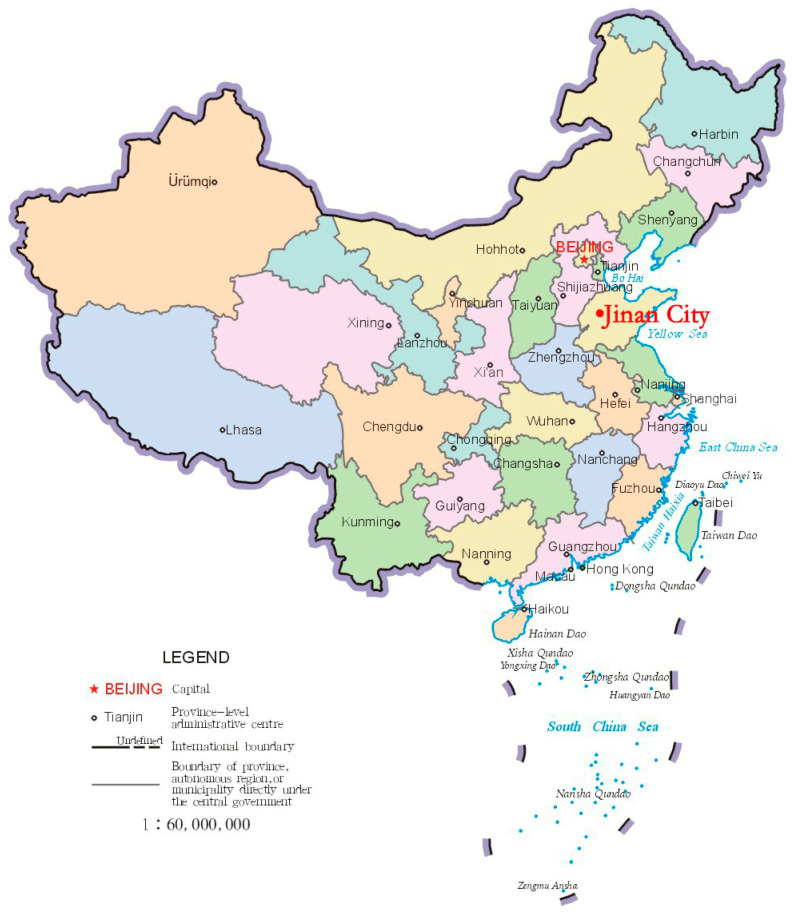
Location map of Jinan City (Form Standard map service: http://bzdt.ch.mnr.gov.cn, accessed on 22 February 2022).

Located in central Shandong Province, Jinan City is the provincial capital and spans a total area of 10,244.45 km^2^ (Figure 1). It is geographically located between 36°02′~37°54′ north latitude and 116°21′~117°93′ east longitude. It belongs to the warm temperate continental monsoon climate zone, with abundant sunlight and four distinct seasons. The average temperature is 14.2 °C, and the annual precipitation is 548.7 mm. According to the Seventh National Census (Department of Statistics Jinan City, 2020), there are 9,202,400 permanent residents in Jinan, with 50.13% males and 49.87% females. The urban population was 6,760,000, or 73.46%, and the rural population was 2,442,425, or 26.54%.

**Figure 2:**
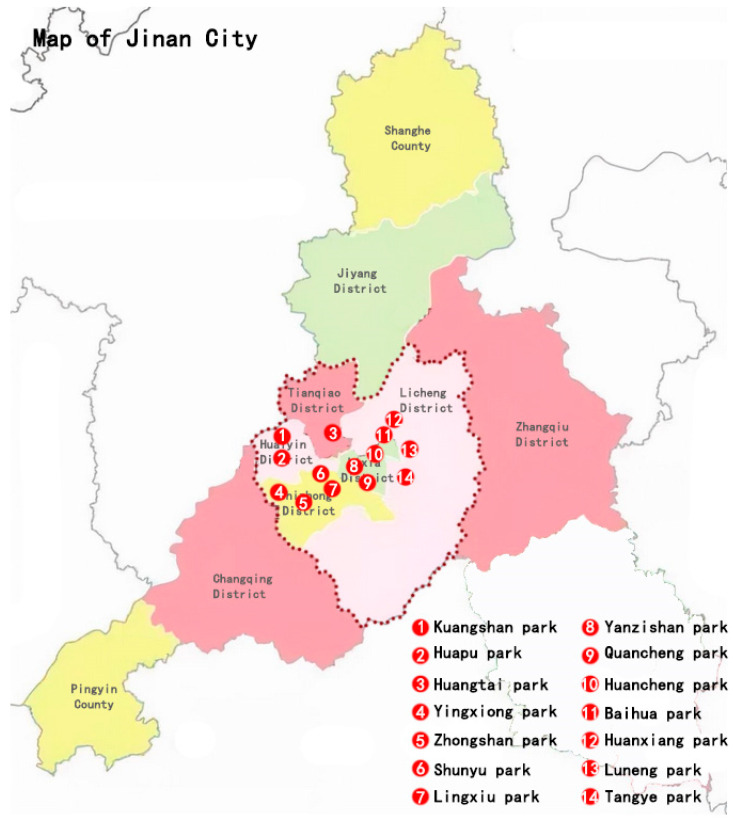
Location map of Jinan community parks.

As shown in Figure 2, Jinan is divided into nine strategic zones, including seven urban districts (Jiyang, Zhangqiu, Shizhong, Lixia, Huaiyin, Tianqiao, and Licheng) and two counties (Shanghe and Pingyin). As the urban population is mainly concentrated in Shizhong, Lixia, Huaiyin, Tianqiao, and Licheng districts, the field research for our study focuses on these five urban districts.

### 2.2. Study Site

There are about 16 community parks identified in the five urban districts in Jinan City. Fourteen representative community parks of similar size were selected, excluding two parks under repair and closed. The remaining 14 community parks and their details are shown in Table 1. The sample community parks are free public parks and open to the public 24 h a day. All the community parks have plants, benches, viewpoints, trails, and activity facilities, and both of them could perform the basic functions of beautiful landscapes and entertainment for residents’ health.

### 2.3. Sample Selection and Design

To gauge the visitor usage patterns and desired improvements to community parks in Jinan City, the respondents were randomly selected in 14 community parks and comprised of workers, retirees, homemakers, children, and so on. Chinese community park visitors of all ages (mainly of Han nationality), obtained through an opportunistic sampling strategy, were included as respondents in this study (M = 42 years old, SD > 0.9). Respondents volunteered for the survey and were not offered compensation. Because of the low-risk nature of the study, including this not being focused on patients or people with specific health conditions, no ethical approval process was required. However, we informed the respondents of the purpose of the study before they agreed to be interviewed, and their verbal consent was obtained. They all agreed that their interview results would be published anonymously.

### 2.4. Survey Instruments and Procedures

This study adopted a mixed research method combining complementary quantitative and qualitative data for collection and analysis. The quantitative data collection component was conducted using interviewer-completed questionnaires, which consisted of four parts: (i) demographic data; (ii) visitors’ usage patterns; (iii) motivations for visiting; and (iv) desired improvements in community parks in Jinan. Meanwhile, qualitative semi-structured interviews provided more detailed data on perceptions and desired improvements among visitors to community parks.

The survey was conducted in December 2019 (winter), March 2020 (spring), July 2020 (summer), and October 2020 (autumn) in the 14 community parks. According to Chinese people’s physiological habits, excluding lunch and dinner time, the surveys were conducted on both weekdays and weekends, in the early morning (6:00–9:00 a.m.), morning (9:00–12:00 a.m.), afternoon (2:00–5:00 p.m.), and evening (7:00–9:00 p.m.) to obtain a sufficiently representative study sample of community park visitors in Jinan City. A total of 550 questionnaires were handed out and eight completed questionnaires were excluded because of missing information. The remaining 542 questionnaires were available. Meanwhile, we divided eight respondents into a group, and the fourth respondent in each group will be interviewed in depth. If the interviewee is not willing to undergo an in-depth interview, the in-depth interview will be postponed to the fifth or sixth place. Based on this, we also conducted 66 in-depth interviews with specific visitors who were willing to engage in detailed discussions. During the interview, the field researcher provided positive feedback to the participants through nods and acknowledgments, and shared the researcher’s personal history, identity, and biases to reassure the respondents.

### 2.5. Analysis

This study used Excel 2210 and SPSS 2.0 software for descriptive analyses of the collected data. The findings are presented and explained quantitatively and qualitatively to determine visitor usage patterns of and desired improvements to urban community parks in Jinan City, China.

## 3. Results and Discussion

Compared to other cultures, our study found several similarities and differences in Chinese perceptions and attitudes about community parks and the improvements desired for their use. To the best of the author’s knowledge, our study is the first to examine the attitudes of Chinese people, especially residents of Jinan City, toward community parks.

### 3.1. Who Uses Community Parks in Jinan City?

#### Demographic Data

As can be seen from Table 2, a total of 542 respondents participated in the survey, including children, students, office workers and retirees, and the majority of them were of Han nationality, which is consistent with the demographic characteristics of Jinan [51]. There were slightly more female visitors than male visitors, which is consistent with the findings of Jasmani et al. [52], Cohen et al. [44] and others. Because female visitors value safety more, community parks meet the conditions of safety, close to home and within their familiar social range, thus they visit them more frequently [12,45]. At the same time, we found in-depth interviews that in addition to safety concerns, additional aspects limited women’s ability to visit parks further away, especially the need for domestic labor, resulting in insufficient time. Nordh and Ostby [39] and Cohen et al. [44] also confirmed that women have highly limited “leisure-time” or “self-time”, whether they are working women, housewives or even retirees.

People over 55 years old accounted for 34.7% and children below 10 years old accounted for 23.6%; these age groups were the main visitors of the community parks. People aged 20–40 made up only 10.3% and they were the least to visit the parks. Because Chinese urban residents over 55 years mainly are retired people who generally fall into two categories. One group is taking care of their grandchildren, and the other is taking care of their fitness, both of which are reasons for older adults to go to the park [12,53,54]. Conversely, young people aged 20–40 are generally under pressure from work, and lacking time is the main factor limiting their access to parks [22,36]. What is noteworthy in our study is that middle-aged people between 40 and 55 years of age also visited the community parks and accounted for 18.8% of the sample. This is novel but understandable, and Veitch et al. [55] in his research of the relationship between physical activity and cognitive function in middle-aged adults, concluded that most middle-aged people have good family and social relationships and relatively stable employment, allowing them to explore their interests, develop and maintain interpersonal relationships, and shift their focus from career to family. The community park can provide them with an open space for family activities and social communication.

### 3.2. How Are the Community Parks Used?

#### 3.2.1. Visitor Usage Patterns

Table 3 shows that 35.4% of respondents go to community parks more than once a day, and 23.6% visit them 3–4 times a week. The most common travel time is 10–20 min (44.6%) and 5–10 min (33.2%), respectively. According to Dunton et al. [25], a community park is more likely to be visited when it is close to one’s residence, and for every 100 m reduction in the distance between home/office and the nearest community park, the probability of visiting the park increases four-fold. The greater the commute from the home/office to a community park, the less frequently it is visited [56,57]. In Shunyu Park, a 58-year-old retired woman also expressed the same opinion:

“*My grandson went to primary school last year, and now I don’t have to look after him all day. So, I go to Shunyu Park, which is close to home and convenient every day, play chess, Tai Chi, and chat with my friends to enjoy time.*”

Furthermore, a total of 52.2% of respondents walk to the park, while 31.0% ride bicycle/e-bike. Few people come to community parks by public transport (14.0%), car (2.2%), or taxi (0.6%). This is understandable as community parks are generally located downtown, where there is much traffic during rush hour, and taking cars or buses and other means of transportation can be time-consuming. Furthermore, a majority of community parks in Jinan are close to residential or office areas and can be easily reached by simple walks. This was also observed in the use of community parks among Danes, where 61% of visits are on foot [45], and in Los Angeles, where about 81% of visits are on foot [44]. According to Neuvonen et al. [27], people prefer to drive or take public transportation to large parks, such as botanical gardens, zoological parks, and amusement parks, on weekends or holidays. This is also confirmed in our study. Most respondents (50.4%) show that they would go to community parks on weekdays, while only a small number mentioned they would go to community parks on public holidays (13.7%) and special events (4.7%). In our in-depth interview, a 28-year-old housewife said,

“*My husband usually drives the family to the zoo or botanical garden outside the city on weekends, but during the week I simply walk with my son to Lingxiu Park for him to socialize and experience nature. This is really convenient.*”

What is remarkable in this study is that 12.2% of respondents visited the park once or twice a month and 11.8% once a year, while most of them were young people aged 20–40. This phenomenon is similar to that of Denver, USA, where young people tend to visit community parks less frequently [58]. According to Chen et al. [36], there are generally two reasons. First, lacking time (being overly busy) is the main reason that constraints young people to visit community parks. The other is that the Internet is more attractive than parks. They rarely go to parks unless they feel they are in suboptimal health [59]. This is true, and the findings of our in-depth interviews confirm this view. According to a 33-year-old male programmer in Tangye park:

“*I prefer to lie down and play the mobile phone or computer games with my friends during my rest time. But since I was diagnosed with moderate fatty liver disease in my physical examination last week, I have decided to run in Tangye Park for an hour after work from now on.*”

Staying at home for prolonged periods has negative effects on both physical (e.g., obesity, high blood pressure, and spinal damage) and mental (e.g., depression and social skills reduction) health [60,61,62]. Attracting people, especially young people, into parks has become vital for both Eastern and Western countries. Many researchers have carried out research and concluded that holding cultural activities in community parks, such as performances and exhibitions, has an extremely positive effect on attracting people to parks [59,63,64]. In addition, Rathore [65] concluded that interesting landscape viewpoints, such as a sea of flowers, exotic art sculptures, and interactive entertainment facilities, are all elements that will attract young people to the park and encourage them to recommend it to their peers.

46.9% of respondents would stay in the community park for 1–2 h, and 40.0% would stay for 30 min–1 h. On the contrary, 3.7% of respondents spent more than 2 h in the community park. This is reasonable, according to the global recommendations on physical activity for the health of the World Health Organization [66], an hour of exercise a day is not only in line with people’s physiological habits but also extremely beneficial to physical health. Our survey confirmed that Chinese residents have a good health concept of “exercising one hour a day, reaping happiness a life”, and the average time the respondents stayed in community parks was 1 h. In addition, our study found that a small number of respondents would stay in community parks for 2 h or more, mostly because of park activities being held, such as charity sales and performances. In line with the ideas of Veitch [55,67] park activities affect the frequency and length of park visits. It can be concluded that positive correlations between “socializing” and “park activities” indicate that these events also provide more opportunities for people to meet and socialize. In addition, there is one thing worth noting in the study: about 9.4% of respondents spend less than 30 min in community parks, as they usually use community parks as a shortcut to their destination. In the in-depth interview, a 32-year-old male office worker said:

“*I prefer to pass through Luneng No. 7 Park on the way home from the bus station. Compared with the street sidewalk, the park road is quieter and safer during rush hours, without noise and cars.*”

Moreover, a great number of respondents (63.5%) are inclined to visit the community parks in a group, where 66.1% are with friends, and 32.5% tend to visit with family members. This is true regardless of sex, with only a minority tending to visit community parks alone (36.5%). This is similar to previous studies of other Asian cultures, especially Malaysians [40], Turks [68,69] and Italians [70], where the majority of people tend to visit parks accompanied by spouses, friends, children or dogs in a large group. White U.S. visitors, however, were a different story, with about 88% preferring to visit the park alone or with one person [71,72]. This has also been observed in the Netherlands, where they prefer to go to parks in small groups, as couples, or alone [68,73]. In addition, according to Peschardt [45], visitors who want to “socialize” generally go to community parks in a large group, while those who need “rest and recuperation” generally visit alone. At the same time as studying the importance of being alone or with company for the psychological recovery of appreciating different environments, Staats and Hartig [74] found that people enjoy the company of friends in urban environments for a variety of reasons, including safety, but when safety is not an issue; in the absence of a companion, recovery is accelerated. This is also supported by several studies, showing that women tend to visit public parks in the company of family or friends rather than alone because their fear levels are significantly higher than men [48,75,76]. For all that, Gu et al. [37] explored the factors that prevent Chinese people from visiting urban parks, founding that the lack of a companion is one of the factors hindering Chinese from visiting the park. In addition, Chinese females also prefer to visit parks in a group, as they feel safer when accompanied by family members or acquaintances [77].

According to the survey, residents in Jinan prefer to visit community parks at 2:00–5:00 p.m. (41.1%), followed by 9:00–12:00 a.m. (23.1%) and 7:00–9:00 p.m. (19.4%). There is no doubt about it because Jinan City has a moderate monsoon climate, and the temperatures in the early morning and night are lower; fewer people prefer to go out, especially in winter. This is consistent with our field observations. Community parks were least visited in the early morning (6:00–9:00 a.m.), especially in winter, and the main visitors were young people doing morning jogging activities. In the morning (9:00–12:00 a.m.), community parks ushered in a small peak of people to visit. This is because the old people entered the park doing tai chi, singing, and other recreational activities. In the afternoon (2:00–6:00 p.m.), community parks had the most visitors, including children, young people, middle-aged people, and old people, and all kinds of activities, such as chatting, sunbathing, and exercise, were taking place. However, the most common activity is interaction, including adult-adult interaction, adult-child interaction, and child–child interaction. In the evening (7:00–9:00 p.m.), the flow of people in the community park dropped, mostly middle-aged women gathering in brightly lit areas in the park for square dancing. This is somewhat different from Western or other cultures, such as India [12], Japan [78], and the United States [79]. The vast majority of women do not prefer going to a park at night; the lack of safety is the main limiting factor. On the contrary, 68% of women prefer to go to urban parks in the evening with a companion in China [80]. In our in-depth interview study, a 56-year-old retired female confirmed this view. She said:

“*I go to Huangtai Park with five or six neighbors every night to dance regularly. The park is near my home, convenient, safe and enjoyable. Now our dancing team has grown to about 20 people, the dynamic music attracting many youthful people to join us in square dancing.*”

In China, community parks generally can bring Chinese security even in the evening. The main reason is that in 1994, the Ministry of Public Security of the People’s Republic of China issued the Urban People’s Police Patrol Regulations [81], stipulating that community police must patrol the area under their control every four hours, which ensures urban and community security to a large extent. Therefore, Chinese residents are willing to go to community parks for recreation in the evening. The findings are directly in line with previous findings that both males and females are more likely to visit parks when they feel comfortable and safe [82]. Crenshaw [83] also concluded in her study that many women praised the lack of harassment and adequate safety as positive attributes that promoted their visits to parks.

#### 3.2.2. Motives for Visiting

**Figure 3:**
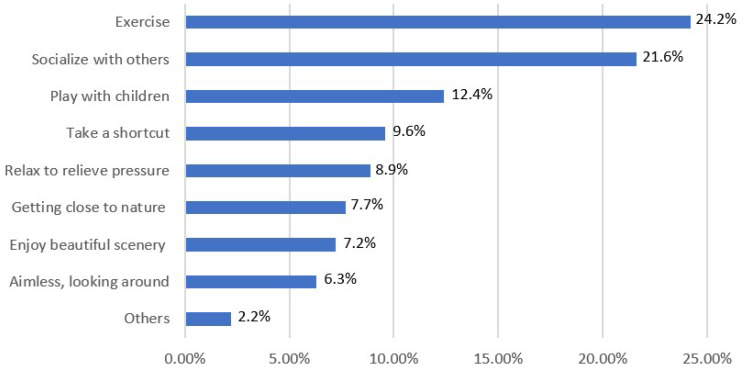
Motives for visiting community parks.

As shown in Figure 3, Chinese urban residents visit community parks for a variety of motives, among which the biggest motivation is “to exercise” (24.2%). This is similar to the situation in other countries [84,85]. This demonstrates the significance of these minor green spaces in the city, where they provide residents with free places to exercise conveniently [31,43,45]. It is not surprising that people use community parks mostly for “exercise” in China. That is largely because community parks, as a form of public welfare for urban residents, do not charge entrance fees and are equipped with free fitness equipment (e.g., spacewalk machines, hip twisters, and tai chi hand presses) sponsored by the Chinese Sports Lottery. These conditions encourage residents to use community parks for exercise. Respondents also visited community parks to “socialize with others” (21.6%), and some visited to “play with children” (12.4%). Others use the park to “take a shortcut” (9.6%), to relax and relieve pressure (8.9%), to get close to nature (7.7%), to enjoy beautiful scenery (7.2%), as well as for aimless (6.3%). This shows the benefits of community parks for people. Ward-Thompson et al. [86] also concluded in their study that urban green spaces such as community parks have far-reaching health benefits for residents, including increased sociability and reduced stress. Moreover, it was further stressed that being in a green environment has a positive effect on people of all ages. Lin et al. [87] and Jasmani et al. [52] also reported that natural features in green spaces have some highly beneficial effects, including reducing stress and improving moods. They found that residents prefer to live surrounded by community parks that can play an active role for them. This was echoed in our in-depth interview according to a 26-year-old office worker:

“*I used to play in the mall with friends, but now we also choose to go to a nearby community park to enjoy nature and take a fresh breath, which makes us cheerful; after all, we always stay inside day and night.*”

### 3.3. What Are the Visitors’ Desired Improvements to the Community Parks in Jinan?

#### Desired Improvements

As shown in Table 4, most respondents (65.7%) were satisfied with community parks in Jinan City and thought only a small amount of improvements were needed. Some respondents (23.8%) were not satisfied and thought many improvements were needed. For the rest, 8.3% of respondents were very satisfied, and 2.2% were very dissatisfied.

In terms of the desired improvements of community parks, most respondents (13.3%) mentioned the addition of physical training facilities, activity areas (7.6%), activity facilities for children (6.8%), dog/pet activity area (4.8%), and bathroom (4.2%). The findings indicate that there is a short supply relationship between urban residents and available resources in community parks, which is in line with the findings in other countries such as America [88], Denmark [89], Indonesia [90] and Thailand [91]. This is real, and we also observed this in the field, e.g., people lining up to use exercise equipment, square dancers interacting with chess players in the same space, and only one sandpit in the park for children to play. A big reason for this result is community parks’ poor planning and design. Vierikko et al. [92] expressed that the suitable space scale of community parks used to guide and control plays a crucial role; residents’ activities are also affected by environmental constraints, and how to provide high-quality activity guide users to use the park, healthy, comfortable environment is a designer should be considered. Kruize et al. [85] and Lin et al. [87] also concluded that the function of the community park layout needs to meet the age people use at the same time, should not only provide an open space but also provide a certain space for a particular activity, ensure security and relatively independent activities, make the community park can meet the demand of many groups, multi-type leisure activities. Williams et al. [93] added that the road system is the core of community parks. Smooth and reasonable park roads can not only serve as the basis for activities such as walking and running but also connect each activity node and organically connect each space. Basu and Nagendra [12] believe that the solution to the problem is to analyze and summarize the core functional needs of a high frequency of daily leisure activities of visitors by studying their usage patterns and desired improvements to urban community parks so as to guide the setting of leisure functions of community parks in the practice process. This is consistent with our research in Jinan City, which emphasizes that the designer of the park should fully understand the surrounding community environment and demographic composition, consider the setting of the park project from the perspective of users, arrange targeted leisure activities and functional sites, and then carry out the overall layout of community park space.

Furthermore, some respondents mentioned increasing landscape viewpoints (3.3%), art sculptures (3.9%), and vegetation (8.3%), which are related to the beauty of the park. Others mentioned keeping park cleanliness (4.4%), concession stands (2.8%), preventing unfavorable visitor behaviors (2.4%), and adding recycling bins (1.5%), which relates to the management and maintenance of community parks. According to the research of Nordh et al. [39,43] and van den Berg [94], the beauty degree of community parks is positively correlated with the participation of visitors. The research found that park “softscape” was positively correlated with access, while park visitors like parks with abundant “softscapes”, such as trees, shrubs, flowers, and water features, but do not want parks with too numerous “hardscapes”, such as stairs, roads, and buildings.

Based on the survey findings, the desired improvement, including emergency buttons (7.6%), lighting (6.5%), rest seats (6.3%), wheelchair accessibility (5.0%), information/interpretive signs (3.1%), and level off the road (2.3%). These are related to community park friendliness. In the in-depth interview, a 74-year-old retired professor from Shunyu Park said:

“*Community parks are usually crowded with elderly people and children, who are more likely to have accidents, thus, the friendliness of the park is very important. It would be better if emergency equipment were installed in the park’s activity areas.*”

Previous studies have shown that people with poor physical and/or mental status are less inclined to visit community parks due to various limitations, but when parks become more friendly and accessible, they are more willing to visit them, which significantly improves their physical and/or mental status [95,96]. Indeed, taking the findings together, our study concludes that individuals with wheelchairs and/or other special needs need greater friendliness and accessibility to community parks. Finally, respondents were also asked if they would use the parks more if changes were made to make community parks more attractive, cleaner, and friendlier. A majority of respondents (79.9%) responded positively.

## 4. Conclusions

An investigation into urban community parks is a relevant topic today, especially for developing countries such as China that are growing rapidly. Studies on visitor usage patterns and desired improvements to urban community parks in the context of Chinese society are imperative in diversifying and growing the field of local knowledge. Whether from the view of the effect of planning a community park itself or the development needs of the current urban renewal, it is necessary to experience and study the community park from the perspective of visitors’ life cognition and habit preference. Our study uses a combination of quantitative and qualitative analysis to clarify who uses the urban community parks and how, and what their desired improvements toward 14 community parks in Jinan City, China, are. This is helpful for the development of community parks. On the one hand, this highlights the importance of community parks in the daily lives of urban residents. On the other hand, it could be helpful to develop strategies to meet the needs of visitors when planning and designing or redesigning and upgrading community parks. This will ensure that community parks are not displaced by urbanization.

However, this study also has some limitations. Our study was conducted during the COVID-19 pandemic. According to China’s epidemic prevention policy on access to public spaces, people should provide a 48 h negative nucleic acid certificate and take their temperature when entering the park. These controls somewhat reduced the frequency with which residents visited community parks, resulting in an incomplete sample size. In addition, it is not a complete study; it represents only selected respondents in community parks and cannot represent all city residents in Jinan City. Furthermore, the field researcher may have had personal biases in selecting respondents, such as unconsciously selecting the same number of men and women and favoring interviews with retirees, which is a potential source of error. We, therefore, suggest that further research should involve more researchers and a larger number of interviews with urban residents (including both visitors and non-visitors to community parks) and expand the sample size to obtain more comprehensive data on community parks. Such research could better prepare for the construction, enhancement, and redevelopment of future urban community parks.

## Figures and Tables

**Table 1:** The community parks in Jinan City.

Number	Community Park Real Photos	Community Park Details
1	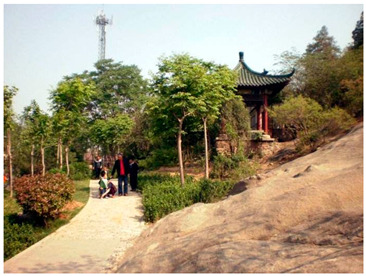	Name: Kuangshan park Size: 6.9 hm^2^ District: Huaiyin district Street name: Jiqing Street GPS location: E 116.954878° N 36.691597°
2	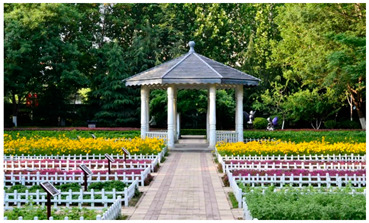	Name: Huapu park Size: 5.8 hm^2^ District: Huaiyin district Street name: Jingshi Street GPS location: E 116.949529° N 36.659509°
3	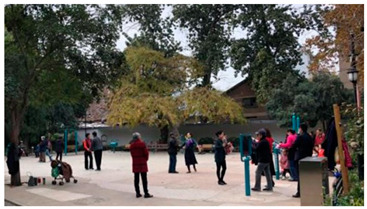	Name: Huangtai park Size: 4.7 hm^2^ District: Tianqiao district Street name: Hangyun Street GPS location: E 117.055937° N 36.711579°
4	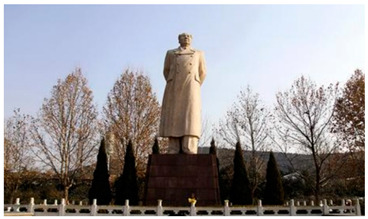	Name: Yingxiong park Size: 7.2 hm^2^ District: Shizhong district Street name: Yingxiongshan Street GPS location: E 117.010969° N 36.645278°
5	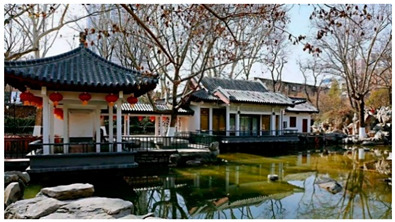	Name: Zhongshan park Size: 6.3 hm^2^ District: Shizhong district Street name: Jingsan street GPS location: E 116.995771° N 36.667601°
6	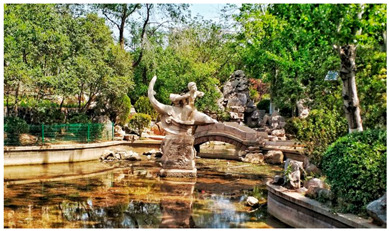	Name: Shunyun park Size: 4.5 hm^2^ District: Shizhong district Street name: Shunyu Street GPS location: E 117.022598° N 36.633229°
7	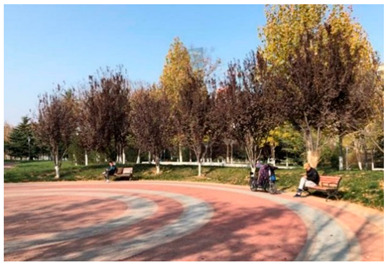	Name: Lingxiu park Size: 4.1 hm^2^ District: Shizhong district Street name: Jianxiu Street GPS location: E 117.009673° N 36.597034°
8	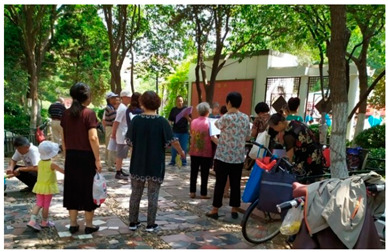	Name: Yanzishan park Size: 4.2 hm^2^ District:Lixia district Street name: Peace Street GPS location: E 117.06713° N 36.663794°
9	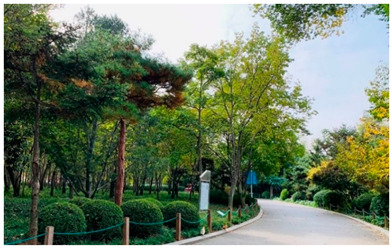	Name: Quancheng park Size: 7.1 hm^2^ District: Lixia district Street name: Jingshi Street GPS location: E 117.026361° N 36.651235°
10	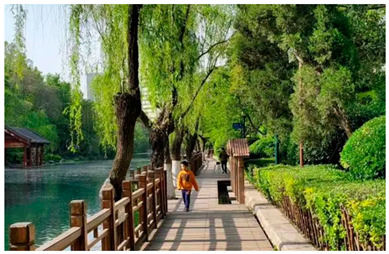	Name: Huancheng park Size: 6.4 hm^2^ District: Lixia district Street name: Jiefang Street GPS location: E 117.038033° N 36.668754°
11	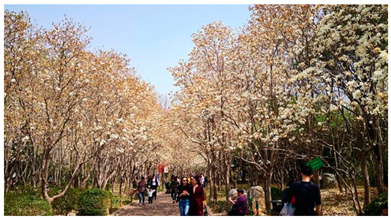	Name: Baihua park Size: 6.9 hm^2^ District: Licheng district Street name: Minziqian stret GPS location: E 117.07713° N 36.681782°
12	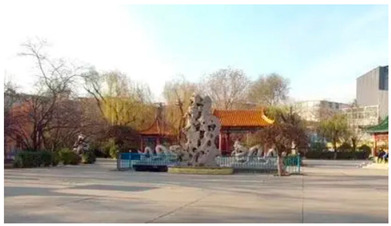	Name: Huanxiang park Size:4.5 hm^2^ District: Licheng district Street name: Huanxiangdian Street GPS location: E 117.083152° N 36.719796°
13	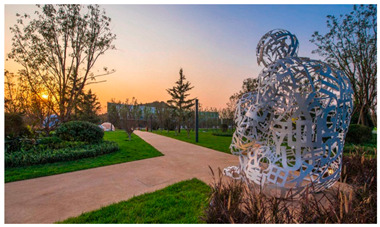	Name: Luneng park Size: 5.3 hm^2^ District: Licheng district Street name: Shiji Street GPS location: E 117.21927° N 36.698993°
14	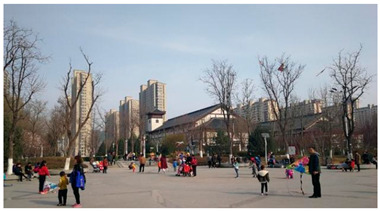	Name: Tangye park Size: 6.5 hm^2^ District: Licheng district Street name: Tangye East street GPS location: E 117.235363° N 36.686717°

**Table 2:** Demographic information of the respondents.

Respondent Information	Total	
	Number (n)	Percentage (%)
Sex?		
Male	264	48.7
Female	278	51.3
Race?		
Han nationality	510	94.1
Hui nationality	32	5.9
Age group?		
Under 10 years	128	23.6
10–20 years	68	12.5
20–40 years	56	10.3
40–55 years	102	18.8
Over 55 years	188	34.7
Occupation?		
Children (≤6 years)	101	18.6
Student	66	12.2
Public sector	64	11.8
Private sector employees	44	8.1
Self-employed	29	5.4
Pensioner	212	39.1
Unemployed	26	4.8

**Table 3:** Analysis of visitor usage pattern.

The Usage Pattern of Visitor	Total	
	Number (n)	Percentage (%)
Would you like to visit this community park alone or in a group?		
Alone	198	36.5
In a group	344	63.5
If in a group, with whom?		
Friends	358	66.1
Family members	176	32.5
Others	8	1.4
How often do you visit this community park?		
≥1 a day	192	35.4
3–4 times a week	128	23.6
1–2 times a week	92	17.0
1–2 times a month	66	12.2
1–2 times a year	64	11.8
How did you get to the park?		
Walk	283	52.2
Bicycle/E-bike	168	31.0
Public transport (bus, subway)	76	14.0
Car	12	2.2
Taxi	3	0.6
How long is the road travel time?		
≤5 min	48	8.9
5–10 min	180	33.2
10–20 min	242	44.6
20–30 min	56	10.3
≥30 min	16	3.0
When would you like to come to this community park?		
Weekdays	273	50.4
Weekends	169	31.2
Public holiday	74	13.7
Special event	26	4.7
What time would you like to visit this community park?		
6:00–9:00 am	89	16.4
9:00–12:00 am	125	23.1
2:00–6:00 pm	223	41.1
7:00–9:00 pm	105	19.4
How long do you normally stay at the park?		
≤30 min	51	9.4
30 min–1 h	217	40.0
1–2 h	254	46.9
≥2 h	20	3.7

**Table 4:** Analysis of visitors’ desired improvements.

Park Desired Improvements	Total	
	Number (n)	Percentage (%)
Are you satisfied with the community park?		
Very satisfied	45	8.3
Satisfactory, fewer improvements are needed	356	65.7
Unsatisfactory, more improvements are needed	129	23.8
Very unsatisfactory	12	2.2
What is the main desired improvement of you to the community park?		
No changes	32	5.9
Landscape viewpoints	18	3.3
Art sculptures	21	3.9
Vegetation	45	8.3
Unfavorable visitor behaviors	13	2.4
Park cleanliness	24	4.4
Add recycling bins	8	1.5
Add bathroom	23	4.2
Add concession stands	15	2.8
Lighting	35	6.5
Emergency buttons	41	7.6
Information/interpretive signs	17	3.1
Level off the road	13	2.4
Wheelchair accessibility	27	5.0
Add rest seats	34	6.3
Add activity area	41	7.6
Add activity facilities for children	37	6.8
Add physical training facilities	72	13.3
Add dog/pet activity area	26	4.8
Would you like to use community parks more often if changes were implemented?		
Yes	433	79.9
Maybe	64	11.8
No	23	4.2
No response	22	4.1

## Data Availability

Not applicable.
